# Effect of electrical grade glass fibres and silver nanoparticles on the mechanical properties of provisional PMMA material

**DOI:** 10.1038/s41598-025-98145-2

**Published:** 2025-04-23

**Authors:** Rohan Yatindra Vaidya, I. N. Aparna, Gayathri Krishnamoorthy, Aditi Chopra

**Affiliations:** 1 Department of Prosthodontics and Crown & Bridge, Manipal College of Dental Sciences, Manipal Academy of Higher Education (MAHE), Manipal, Karnataka 576104 India; 2 Department of Periodontology, Manipal College of Dental Sciences, Manipal Academy of Higher Education (MAHE), Manipal, Karnataka India

**Keywords:** Antimicrobial Provisional restorations, Long term Provisional Restoration, Long Span Provisionals, AgNPs reinforced PMMA, E-Glass reinforced PMMA, Health care, Medical research

## Abstract

The use of provisional crowns and bridges rendered the necessary care for the prepared teeth. The protection of the prepared tooth is one of the most important factors in the long-term success of fixed dental prosthesis. Temporary crowns and bridges for longer periods of use are most often made of acrylic material. Unfortunately, it does not have the appropriate mechanical properties or resistance to microbial colonization Therefore, the purpose is to modify it by adding glass fibers (2% w/w) and silver nanoparticles (0.5% w/w). A total of 160 samples were prepared which were segregated into 4 groups based on the test performed. Each group had 4 subgroups which consisted of samples containing silver nanoparticles (0.5% w/w) and E-glass fiber (2% w/w) mixed with PMMA in their respective concentrations. The samples were then tested for surface roughness, micro-hardness, flexural strength and SEM Analysis. The results of the study showed that there were no effect on the surface roughness values after incorporating silver nanoparticles and E-glass fibers. However, samples containing silver nanoparticles and E-Glass fibre individually had higher values of microhardness and flexural strength than those who had both together. The SEM images showed clumping of silver nanoparticles non-uniform orientation of E-glass fibers. Thus, it can be concluded that silver nanoparticles and E-glass fibre when added separately to PMMA, enhance its mechanical properties. However, better methods of mixing PMMA with silver particles and glass fibers is needed to attain a uniform distribution. Further, the orientation of E-glass fibers could also have an effect on the flexural properties of PMMA.

## Introduction

The protection of a prepared tooth intraorally is one of the key factors that determines the long-term success of a fixed prosthesis. The placement of a provisional restoration over a prepared tooth protects against thermal stimuli, bacteria and mechanical injuries. Provisional restorations generally belong to two groups, i.e., acrylic resins (polymethyl methacrylate (PMMA) and composite resins, with the latter having superior mechanical properties. However, as PMMA possesses certain key advantages over its rival in being economical, having higher elasticity and being easily repairable, it has gained popularity. From being used exclusively as a denture base material by Walter Wright in 1937^1^, acrylic resin soon started being used as a provisional material. However, it has several drawbacks compared with its counterpart (composite resins), such as a high coefficient of thermal expansion, substandard mechanical strength, brittleness, and low modulus of elasticity^2^. All these shortcomings of acrylic make its use questionable. In addition, in certain situations or conditions, provisional restorations must be used for an extended duration. In such unforeseen events, provisional restorations need to remain dimensionally stable intraorally under occlusal loading and possess some amount of antimicrobial activity to fight oral microorganisms.

A study performed by Ankita Singh and Sandeep Garg in 2016 revealed that the flexural strength of provisional restorations decreases over time^3^ Thus, to ensure that the integrity of the material is intact, PMMA has been reinforced with various additives. A variety of techniques have been described, including the incorporation of fibres (glass fibres, carbon/graphite fibres, ultrahigh-molecular-weight polyethylene (UHMWP), and aramid fibres^4,5^, microcrystalline cellulose, and metal nanoparticles, among others, to enhance the mechanical properties.

When placed intraorally, the prosthesis is exposed to a variety of microbial flora, ranging from gram-positive bacteria to gram-negative bacteria and fungi^6^. The antimicrobial property of PMMA has been rendered mainly through the addition of foreign materials (synthetic substitutes such as silver nanoparticles (AgNPs) or natural substitutes such as neem)^7^. AgNPs have chemical, physical, and biological capabilities that are significantly better than those of outdated bulk materials because of their nano size. They are also known to improve a material’s mechanical properties.

Numerous studies have investigated the interactions between E-glass fibres and silver nanoparticles with PMMA. Each of these combinations has been shown to increase the strength of the material individually. However, PMMA reinforced with E-glass fibres does not show any antimicrobial activity^7^. Thus, the current study aims to assess the synergistic effect of incorporating silver nanoparticles along with E-fibres into PMMA in an attempt to enhance the mechanical properties and enhance the antibacterial properties of the material.

## Materials and methods

### Material description

Tooth-coloured self-polymerising acrylic resin (DPI tooth molding resin: polymer and monomer, dental product of India: Bombay Burmah Trading Corporation Ltd.) was mixed with the desired quantity of Silver Nanoparticles (Nano Research Labs, Jamshedpur, India) and E-Glass fibres (Goa Glass Factory, Goa, India) to prepare the test samples [Table 1].

**Table 1:** Details of the various groups and subgroups along with their descriptions.

Group No	Group description	Subgroup number	Subgroup description
Group 1 (G1)	Surface Roughness	Subgroup 1A (SG1A)	PMMA
		Subgroup 1B (SG1B)	PMMA + AgNps
		Subgroup 1C (SG1C)	PMMA + E-Glass Fibre
		Subgroup 1D (SG1D)	PMMA + AgNps + E-Glass
Group 2 G2	Micro-Hardness	Subgroup 2A (SG2A)	PMMA
		Subgroup 2B (SG2B)	PMMA + AgNps
		Subgroup 2C (SG2C)	PMMA + E-Glass Fibre
		Subgroup 2D (SG2D)	PMMA + AgNps + E-Glass
Group 3 G3	Flexural Strength	Subgroup 3A (SG3A)	PMMA
		Subgroup 3B (SG3B)	PMMA + AgNps
		Subgroup 3C (SG3C)	PMMA + E-Glass Fibre
		Subgroup 3D (SG3D)	PMMA + AgNps + E-Glass
Group 4 G4	Particle Distribution	Subgroup 4A (SG4A)	PMMA
		Subgroup 4B (SG4B)	PMMA + AgNps
		Subgroup 4C (SG4C)	PMMA + E-Glass Fibre
		Subgroup 4D (SG4D)	PMMA + AgNps + E-Glass

### Material specification

- Powder – (Polymer – Polymethylmethacrylate; Pigments—non-toxic pigments [Shade C])
- Liquid – (Monomer – Methyl methacrylate; Initiator – Benzoyl Peroxide)
- E-Glass Fibers – Pre silanized glass fibers of diameter 20microns were selected and then sectioned into smaller fragments of 2 mm length (the fibers were first measured using a scale and then cut)
- Silver Nanoparticles – Size 30-50 nm, spherical shape, specific surface – 15–20 m^2^/g

### Estimation of silver nanoparticle and E-Glass fibre concentrations

The concentrations of silver nanoparticles and E-glass fibres were assessed in prior studies performed by different authors^7–9^. The concentration of silver nanoparticles used for attaining a perfect blend of mechanical and antibacterial properties was ascertained from the work of Kassaee et al.^10^, which was 0.5% (w/w), whereas a 2% weight percentage of E-glass fibres combined with acrylic resin was derived from the work of Nagpal et al.^9^.

#### Testing procedures and parameters

##### Test sample specification

An aluminium mould was used for the fabrication of the test samples. The dimensions of the test samples were in accordance with the International Standards Organisation Specification (ISO) No. 10477:2020 Dentistry – Polymer-based crown and veneering material.

##### Test sample fabrication

Subgroup 1 (PMMA) *[n* = *40]*

The first subgroup consisted of only tooth-coloured autopolymerising acrylic resin. The monomer and polymer were dispensed in the appropriate ratio (3:1 – powder : liquid) as specified by the manufacturer and mixed by hand via a mixing spatula for 30 seconds.

Subgroup 2 (PMMA + AgNPs) *[n* = *40]*

In the second subgroup, a mixture of acrylic resin and silver nanoparticles was formulated. Depending on the weight of the acrylic polymer, an appropriate amount of silver nanoparticles (0.5% w/w) was measured via a microweighing scale. The same quantity of acrylic powder was removed before the silver nanoparticles were added to the powder. . Once added, the acrylic powder was thoroughly mixed manually. Once the acrylic and silver nanoparticle mixture was ready, the monomer and polymer were dispensed at the appropriate ratio as specified by the manufacturer and mixed.

Subgroup 3 (PMMA + E-Glass Fibres) *[n* = *40]*

The third subgroup contained acrylic powder with a 2% (w/w) concentration of E-glass fibres procured from the Goa Glass factory, Goa, India. The E-glass fibre was cut into sections of length 2mm in lab with the help of a bard parker knife, weighed in accordance with the weight of the acrylic powder and then mixed with the acrylic powder. Once the acrylic and silver nanoparticle mixture was ready, the monomer and polymer were dispensed at the appropriate ratio as specified by the manufacturer and mixed.

Subgroup 4 (PMMA + AgNPs + E-Glass Fibres) *[n* = *40]*

The final test group contained a combination of silver nanoparticles and E-glass fibres mixed at an appropriate concentration with acrylic powder. Once the acrylic and silver nanoparticle mixture was ready, the monomer and polymer were dispensed at the appropriate ratio as specified by the manufacturer and mixed.

In each subgroup, as the acrylic resin reached the dough stage, it was packed into aluminium molds, which were pre-seasoned with petroleum jelly and placed under a dental hydraulic press at 100PSI for 10 min Once the material was set, the hydraulic press was released, and the samples were retrieved. Following retrieval, the samples were left undisturbed for 48 h for the completion of polymerisation after which the samples were trimmed with metal trimming burs and then finished with different grits of sandpaper (180 → 220 → 320 → 600). The finished acrylic samples were then polished with pumice.

#### Testing equipment

- **Surface roughness: **A 2D profilometer (Taylor Hobson – FORM TalySurf 50, United Kingdom) is used for the estimation of surface roughness. The equipment derives the surface roughness value on the basis of the reading obtained by the diamond stylus as it traverses the surface of the sample for a distance of 2.5 mm while exerting a force of 1 mN.
- **Microhardness: **The microhardness was assessed using a Vickers Hardness Tester (Mitutoya – HM200, Japan) The equipment uses a pyramid-shaped indent created on the sample to calculate the hardness value. The diamond indenter creates an indent on the surface of the sample during this test. The Vickers hardness is calculated by the system using formulas on the basis of the diagonal lengths of the indent, which are marked by visualising the indent through a microscope.
- **Particle distribution: **Scanning electron microscope [JOEL Ltd, Tachikawa, Tokyo, Japan] was used to assess the uniformity and distribution (homogenous / nonhomogeneous) of silver nanoparticles and E-glass fibres within the test sample.
- **Flexural strength: **Flexural strength was calculated with the aid of a universal testing machine (UTM) [INSTRON 3366, Norwood, USA]. Each sample was positioned in the UTM on a 20 mm long support and subjected to a 3-point flexural strength test with a crosshead speed of 5 mm per minute until failure. Following this, the values for flexural strength were obtained via the given formula as shown in equation 1.
  1 $$Flexuralstrength = \frac{3F*L}{{b*d^{3} }}$$

F = force at the fracture point

L = support span length

b = width of the sample

d = height of the sample

### Statistical analysis

The statistical software SPSS 26.0 (SPSS Inc., Chicago, IL) was utilised for data analysis, with a significance threshold of p < 0.05. To evaluate the mean and standard deviation of each group, descriptive statistics were used. The Shapiro‒Wilkinson test was used to determine whether the data were normally distributed. The Kruskal‒Wallis test and the Bonferroni post hoc correction were used for inferential statistical analysis to determine group differences.

## Results

### Surface roughness (Group 1)

The lowest mean surface roughness value (Ra) was found in the 1D subgroup [PMMA + AgNPs + E-glass fibre] (0.1307Ra), whereas the highest value was observed in the 1B subgroup [PMMA + AgNPs] (0.1871Ra). However, the lowest and highest Ra values were observed for subgroup 1A [PMMA] (0.04Ra) and subgroup 1B [PMMA + AgNPs] (0.70Ra), respectively (Table 2). There was no significant difference in the POSTHOC Bonferroni correction between the various Ra values of the subgroups.

**Table 2:** Descriptive Details of the Group 1 (Surface Roughness) (Unit – Ra).

Subgroup	N	Mean	Std. deviation	Std. error	95% Confidence interval for Mean		Min	Ma
					Lower bound	Upper bound		
SG1A	10	0.1575	0.16357	0.0517	0.0405	0.2745	0.04	0.54
SG1B	10	0.1871	0.22972	0.0726	0.0227	0.3514	0.05	0.70
SG1C	10	0.1842	0.12535	0.0396	0.0945	0.2739	0.06	0.49
SG1D	10	0.1307	0.12322	0.0389	0.0426	0.2188	0.06	0.44
Total	40	0.1649	0.16130	0.0255	0.1133	0.2165	0.04	0.70

SG1A – Subgroup 1A – PMMA; SG1B – Subgroup 1B – PMMA + AgNPs; SG1C – Subgroup 1C – PMMA + E-glass fibres; SG1D – Subgroup 1D – PMMA + AgNPs + E-glass fibres.

### Microhardness (Group 2)

Among the various subgroups, subgroup 2A [PMMA] had the highest hardness value of 18.0Hv, whereas subgroup 2D [PMMA + AgNPs + E-Glass Fibre] had the lowest value of (12.7Hv). The highest mean hardness values belonged to subgroup 2C [PMMA + E-Glass Fibre] (17.00Hv), whereas the lowest value belonged to subgroup 2D [PMM A + AgNPs + E-Glass] (13.33Hv) (Table 3).

**Table 3:** Descriptive Details of the Group 2 (Microhardness)( Unit – Hv).

Subgroup	N	Mean	Std. Deviation	Std. Error	95% Confidence Interval for Mean		Min	Max
					Lower Bound	Upper Bound		
SG2A	10	15.240	1.3134	0.4153	14.300	16.180	13.9	18.0
SG2B	10	16.070	0.4270	0.1350	15.765	16.375	15.5	16.8
SG2C	10	17.000	0.3742	0.1183	16.732	17.268	16.5	17.8
SG2D	10	13.330	0.4809	0.1521	12.986	13.674	12.7	14.3
Total	40	15.410	1.5500	0.2451	14.914	15.906	12.7	18.0

SG2A – Subgroup 2A – PMMA; SG2B – Subgroup 2B – PMMA + AgNPs; SG2C – Subgroup21C – PMMA + E-glass fibres; SG2D – Subgroup 2D – PMMA + AgNPs + E-glass fibres.

Post hoc Bonferroni correction was carried out to perform multiple comparisons within each subgroup, which revealed a significant difference between the following subgroups:

- PMMA vs PMMA + E-glass Fibre (P < 0.05) (0.000)
- PMMA vs PMMA + AgNPs + E-Glass Fibres (P < 0.05) (0.000)
- PMMA + AgNPs vs PMMA + AgNPs + E-Glass Fibres (P < 0.05) (0.000)
- PMMA + E-glass Fibre vs PMMA + AgNPs + E-Glass Fibre (P < 0.05) (0.000)

### Flexural strength

The highest and lowest flexural strengths were obtained for subgroup 4C [PMMA + E-glass fibre] (147.60 MPa) and subgroup 4 (132.66 MPa), respectively. Similarly, for the mean flexural strength, the highest mean was reported for subgroup 4B [PMMA + AgNPs] (132.99 ± 9.02 MPa), whereas the lowest mean was reported for subgroup 4D [PMMA + AgNPs + E-glass fibre] (109.39 ± 11.92 MPa), as shown in Table 4.

**Table 4:** Descriptive details of Group 3 (Flexural Strength) (Unit – MPa).

	N	Mean	Std. Devi	Std. Error	95% Confidence interval for Mean		Minimum	Maximum
					Lower bound	Upper bound		
SG3A	10	122.1520	11.88285	3.75769	113.6515	130.6525	93.77	133.82
SG3B	10	132.9920	9.02767	2.85480	126.5340	139.4500	120.54	146.46
SG3C	10	132.2730	17.02283	5.38309	120.0956	144.4504	87.69	147.60
SG3D	10	109.3911	11.92508	3.97503	100.2247	118.5575	93.89	132.66
Total	40	124.5818	15.54677	2.48947	119.5421	129.6215	87.69	147.60

SG3A – Subgroup 3A – PMMA; SG3B – Subgroup 3B – PMMA + AgNPs; SG3C – Subgroup 3C – PMMA + E-glass fibres; SG3D – Subgroup 3D – PMMA + AgNPs + E-glass fibres.

For multiple comparisons within groups, the post hoc Bonferroni correction was used, and the results revealed a statistically significant difference between the groups mentioned below.

- PMMA + AgNPs vs PMMA + AgNPs + E-Glass Fibre (P < 0.05) (0.002)
- PMMA + E-glass Fibre vs PMMA + AgNPs + E-Glass Fibre (P < 0.05) (0.003)

### Particle distribution

**Fig. 1:**
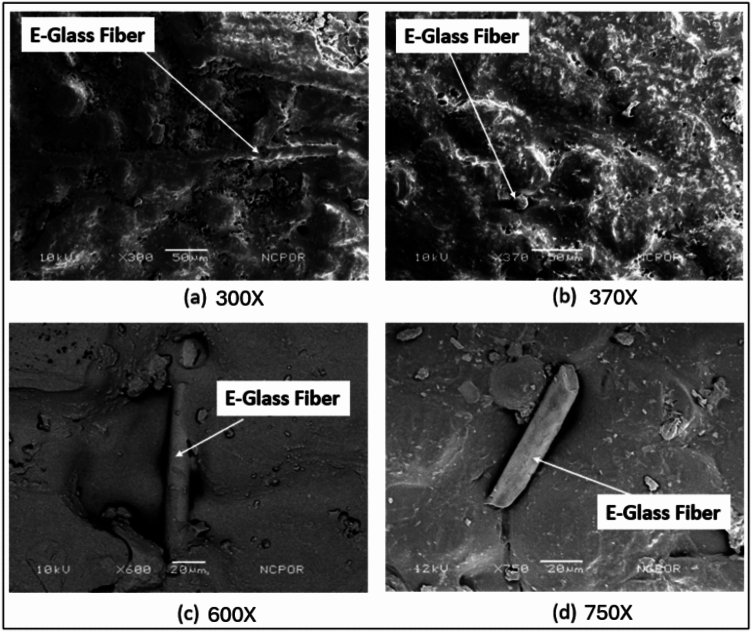
SEM images of the E-glass fibre taken at different magnifications showing different orientations of the fibre strand.

**Fig. 2:**
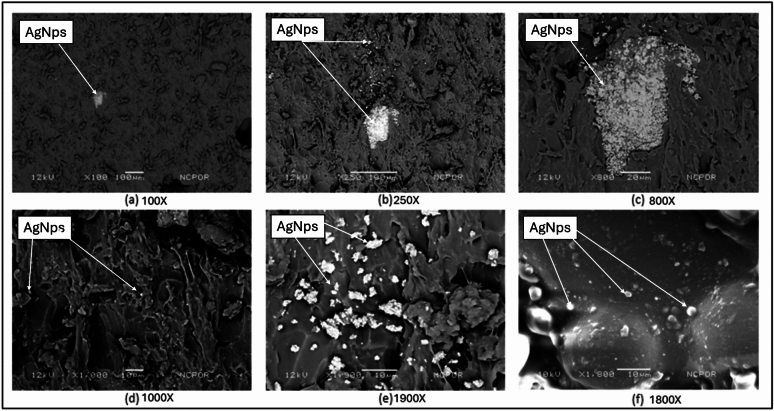
SEM images of silver nanoparticles taken at different magnifications in increasing order from (**a**) → (**f**). Images (**a**), (**b**) and (**c**) show the aggregation of the nanoparticles. Image (d) shows a uniform distribution.

The presence of E-glass fibres and silver nanoparticles within the samples can be observed at different magnifications, as shown in Fig. 1. However, the silver nanoparticles were not homogenously distributed. Both locations where the nanoparticles were found to agglomerate and areas where the distribution was uneven were present (Fig. 2). The E-glass fibres were visible at a lower magnification because of their larger size and were distributed evenly. However, the orientation of the fibres was not uniform.

## Discussion

The teeth in the oral cavity are exposed to various types of environmental influences, both from the consumed food and the mastication forces. The tooth is usually protected by the outer enamel layer, which is one of the most resistant structures. When a tooth is prepared to receive a crown, a significant amount of the tooth structure is removed. In order to protect the tooth from adverse effects, a provisional restoration is placed. The provisional restoration needs to remain dimensionally stable under heavy occlusal loads and needs to stop microbial growth on its surface for an extended duration of time^11–13^. Thus, the material from which a provisional restoration is fabricated is reinforced with fillers. The different types of fillers that are incorporated include carbon fibres, aramid fibres, nylon fibres, rayon fibres and glass fibres. Among the various fibre reinforcements glass fibres have become increasingly popular because of their excellent bonding characteristics with polymers and good aesthetic attributes. Glass fibres are typically made up of alumina, lime, and borosilicate, making them organic materials with a low predisposition to moisture absorption, a high melting point, good thermal stability and good resistance to chemicals, which makes them suitable reinforcement agents^14,15^. As these fibres bond well to polymers, they directly influence the mechanical properties.

In 2013, Rama Krishna Alla et al.^2^ tested various types of fillers to PMMA and concluded that E-glass fibres were the best because of their excellent aesthetic qualities and capacity to link with polymers. The different forms of fibres available include chopped strand mats, woven, continuous, unidirectional, etc., of which the chopped strand mats have shown significant results^16^. Among the various concentrations of glass fibres mixed with PMMA, the 2% w/w mixture exhibited the best mechanical characteristics^9,17^. In addition to the weight percentage of glass fibres, the length and type also have a significant effect on the material.

The oral cavity is filled with different kinds of microorganisms ranging from gram-positive to gram-negative^18^. The protection of the tooth from these microbes is very important because the exposed dentinal tubules can act as pathways for the microorganisms to enter the tooth and cause caries. Owing to the increase in the prevalence of antibiotic-resistant microorganisms, the use of silver-containing antiseptics is becoming increasingly common. Silver is a broad-spectrum bactericidal additive that, when incorporated in the form of nanoparticles, has shown promising results. A study by Sodagar et al.^7^ revealed that the incorporation of 0.05% AgNPs into different brands of PMMA influences the flexural properties of the material. However, Kassaee et al.^10^ reported that the inclusion of 0.5% AgNPs in self-curing acrylic resin increased the flexural strength and provided the material with antibacterial properties. Therefore, the most important factors influencing the mechanical properties are the kind of denture base material utilised, the concentration and characteristics of the silver nanoparticles used, and the polar interactions between the C = O groups of PMMA and the silver nanoparticles.^19^. Another study was performed by Sahana Bajracharya^20^ and colleagues to assess the efficacy of silver nanoparticles against Candida biofilm formation. They conducted tests on several groups with concentrations of 0.5%, 1%, and 1.5% silver nanoparticles. All of these groups had a positive antibacterial effect, and the flexural strength of these materials was within the acceptable range.

The surface characteristics of the two surfaces of a crown are highly different. The inner surface must be rough to enhance the bonding characteristics of the restoration, whereas the outer surface must be smooth to help reduce biofilm formation. Numerous studies in the literature have shown that rougher surfaces promote bacterial adhesion and proliferation, which eventually leads to biofilm formation^21–24^. Thus, if the outer surface of a provisional restoration is rough, it will favour the attachment of microbes and make it difficult to eliminate them. Therefore, any modification of the material to enhance its mechanical behaviour should not cause a significant change in the roughness. In this study, the incorporation of silver nanoparticles and E-glass fibres in tooth-coloured PMMA did not significantly affect the roughness. Although a uniform and standard procedure was followed for trimming and polishing, the test sample (subgroup 4), which contained all three materials (PMMA, AgNps & E-Glass), had the lowest mean surface roughness (0.1307). However, subgroup 1 (PMMA) had the lowest values among all the readings (0.04) compared with 0.06 Ra for subgroup 4 (PMMA + AgNPs + E-Glass). A comparison within each subgroup revealed that there was no significant difference among the various subgroups. In fact, surface roughness is one of the various surface parameters that influence microbial adhesion. A recent review published in 2021 by Zheng et al.^22^ outlined the recent work that has been performed to better understand how surface charge, surface wettability, roughness, topography, stiffness, and a combination of qualities affect bacterial adherence. They concluded that various surface characteristics, bacterial motility, or the hydrodynamic circumstances of the environment affect how bacteria sense and bind to surfaces. Consequently, scientific endeavours evaluating the influence of many surface factors on bacterial adherence are crucial to enhance comprehension, instead of concentrating on a single surface parameter and its impact on adhesion.

. On the basis of their composition, the majority of these materials can be divided into two groups: composite resins and methyl methacrylate resins.^27,28^. Traditional methyl methacrylate-type resins are monofunctional, low-molecular-weight, linear molecules with poor strength and stiffness; in contrast, composite resins are difunctional and able to cross-link with other monomer chains to increase material strength and toughness.^29^. Thus, PMMA is reinforced with materials that enhance its physical properties. However, in the present study, subgroup 3 (PMMA + AgNPs) had the highest mean microhardness at 17.00 Hv. Although subgroup 1, which contained only PMMA, presented the highest reading of 18 Hv, it also presented a large standard deviation because its mean hardness value decreased to 15.24 Hv. The test group that presented the next highest hardness value was the group with E-glass fibre reinforcement (17.8 Hv), followed by the group with silver nanoparticle-reinforced PMMA (16.8). The test sample that contained both PMMA with silver nanoparticles and E-glass fibres had the lowest hardness (12.7 Hv). With a relatively low standard deviation, this group also had the lowest mean hardness among all test groups (13.33 Hv). In a study performed by Tamer H Hamdy in 2024^30^, carbon nanotubes doped with silver were added to self-curing acrylic. With a p value less than 0.05, the modified groups containing Ag-doped CNTs had a significantly greater microhardness—29.7 Hv—than did the control groups (16.4 Hv). The reason for the difference in values is difficult to pin point. There can be numerous possible explanations for the differences which may include material properties, mixing methods, curing parameters, inclusion of impurities, etc.

The flexural strength of the interim material is crucial, especially if a long-span prosthesis is planned, the patient displays parafunctional habits or has to use the temporary restoration for a lengthy amount of time. Currently, none of the provisional restorations on the market meet the optimal requirements for all situations^31^. The chosen product is the choice of clinicians, which is based on ease of manipulation, cost and aesthetics^29^.. The flexural properties of the various test groups also exhibited results similar to those of the microhardness, where test subgroup 3 had the lowest mean flexural strength. The mean values for the flexural strength of subgroups 2 and 3 were very similar at 132.99 ± 11.88 MPa and 132.27 ± 17.02 MPa, respectively.

As seen from the test results, the microhardness and flexural strength of the group containing both E-glass fibres and AgNPs were significantly lower than those of the other subgroups that contained both AgNPs and E-glass fibres. This difference in the mechanical properties can be attributed to the dispersion of silver nanoparticles and E-glass fibres within the acrylic sample. As shown in the SEM images (Fig), the silver nanoparticles aggregate rather than disperse uniformly. Thus, these regions where we find silver aggregation may act as weak points due to improper monomer‒polymer interactions of the acrylic. This could lead to lower values of the mechanical parameters. Unlike silver nanoparticles, the E-glass fibres did not clump, forming isolated areas. They were dispersed evenly throughout the acrylic. Although the distribution of the glass fibres was even, the orientations of the fibres were different. This difference in orientation could have possibly led to the decrease in flexural strength. For a test sample to have high flexural strength, the glass fibres must be aligned parallel or perpendicular to one another in one plane, and the plane in which the glass fibres are arranged must be perpendicular to the direction of the face. Various other studies have reported similar results and have tried to explain the possible reasons for these results. One of the earlier studies was performed by Stipho and colleagues in 1998, who reported that the decrease in strength may be due to the clumping of glass fibres, which eventually leads to an unequal distribution of the fibres throughout^17^. In 2008, Xia et al.^32^ reported that silver can act as an impurity that can decrease the mechanical strength of a material. Studies have even suggested that the presence of unreacted monomers within the samples could be a possible reason for the lower mechanical parameter values^32,33^. On the other hand, when we observe the values of the subgroups where the E-glass fibres and silver nanoparticles were added separately to PMMA, the readings are much greater than those of the control group, which contained only PMMA. The increase in flexural strength upon the addition of E-glass fibres can be attributed to the work of Vallitu et al.^34^, who reported that the increase in strength could be due to better bonding between the fibres and the resin.

The results obtained in this study showed that E-glass fibres and silver nanoparticles are suitable materials for reinforcement, as stated by different authors. However, the addition of both silver nanoparticles and E-glass fibres to achieve mechanical advantages and antibacterial effects needs further evaluation. Although all the values attained for microhardness and flexural strength are much greater than the minimum requirements set by the ISO, chairside mixing of silver nanoparticles and E-glass fibres does not disperse the nanoparticles evenly. Additionally, the orientation of the E-glass fibres within the samples is an important aspect that needs to be assessed and analysed to further enhance the mechanical properties.

## Conclusion

This study saw the testing of four different materials which consisted of 4 main ingredients in various concentrations. The primary reason for combining both, Silver nanoparticles and E-glass fibers with PMMA was to attain better mechanical and antimicrobial properties. However, it was seen clearly that the mechanical properties got affected in the process. Better methods to attain homogeneity and fiber orientation are necessary for further testing.

### Limitations and future scope

The present study was performed with utmost care and precautions. However, certain errors are inevitable. Owing to the use of different materials and techniques, some human error can occur. The concentrations of E-glass fibres (2%) and silver nanoparticles (0.5%) were assessed on the basis of the available literature. Other researchers reported that these concentrations are biocompatible, nontoxic and favourable for in vitro studies. The drawbacks present in those studies, however, were not taken into consideration. The dispersion of silver nanoparticles within the PMMA samples was not uniform according to the SEM analysis. This uneven dispersion could be a reason for the lower mechanical properties. The best methods for combining silver nanoparticles with PMMA need to be considered. The orientation of the E-glass fibres plays an important role in the flexural strength of the material. As shown in the SEM images, the E-glass fibres were not uniformly oriented. The orientation of the glass fibres must be such that they should lie perpendicular to the direction in which the force is applied. This kind of orientation provides more resistance to flexion.

### Future scope

- Studies focusing on the orientation of glass fibres need to be carried out.
- Better mixing equipment/techniques for PMMA and silver nanoparticles.
- Newer technologies such as 3D-printed materials, which allow layer-by-layer deposition of materials, need to be considered.
- More mechanical parameters, such as compressive strength, can be assessed on tooth samples to better understand the material behaviour.

## Data Availability

The datasets used and/or analysed during the current study are available from the corresponding author on reasonable request.
